# *N*-Heterocyclic Carbene Copper (I) Complexes Incorporating Pyrene Chromophore: Synthesis, Crystal Structure, and Luminescent Properties

**DOI:** 10.3390/molecules28104025

**Published:** 2023-05-11

**Authors:** Yaping Cheng, Geoffrey Gontard, Abderrahim Khatyr, Michael Knorr, Hani Amouri

**Affiliations:** 1 Institut Parisien de Chimie Moléculaire (IPCM), UMR CNRS 8232, Sorbonne Université-Campus Pierre et Marie Curie, 4 Place Jussieu, CEDEX 05, 75252 Paris, France; yaping.cheng@sorbonne-universite.fr (Y.C.); geoffrey.gontard@sorbonne-universite.fr (G.G.); 2 Institut UTINAM, UMR CNRS 6213, Université de Franche-Comté, 16 Route de Gray, 25030 Besançon, France; abderrahim.khatyr@univ-fcomte.fr (A.K.); michael.knorr@univ-fcomte.fr (M.K.)

**Keywords:** luminescent copper complexes, *N*-heterocyclic carbenes, blue emitters, X-ray structural determination

## Abstract

Luminescent *N*-heterocyclic carbene chloride copper (I) complexes incorporating pyrene chromophore (1-Pyrenyl-NHC-R)-Cu-Cl, (**3**, **4**) have been prepared and fully characterized. Two complexes were prepared with R = methyl (**3**) and R = naphthyl groups (**4**) at the nitrogen center of the carbene unit to tune their electronic properties. The molecular structures of **3** and **4** have been elucidated by X-ray diffraction and confirm the formation of the target compounds. Preliminary results reveal that all compounds including the imidazole-pyrenyl ligand **1** are emissive in the blue region at room temperature in solution and in solid-state. All complexes display quantum yields comparable or higher when compared to the parent pyrene molecule. Interestingly replacement of the methyl by naphthyl group increases the quantum yield by almost two-folds. These compounds might show promise for applications as optical displays.

## 1. Introduction

*N*-heterocyclic carbenes (NHCs) are strongly coordinating ligands and have proven themselves as the ligands of choice in a wide range of the chemistry spectrum spanning from organometallics, catalysis to medicinal chemistry [1,2,3,4,5]. More recently they have been used with success to design stable luminescent organometallic and coordination complexes [6,7,8,9]. This is because they tend to push the ^3^dd dark states high in energy and thus avoid the deactivation processes of the low-lying MC (metal-centered) transition states. In a previous work, we demonstrated that cyclometalated octahedral iridium complexes containing NHC-NI (NI = Naphthalimide) are strongly phosphorescent emitters in the red to near IR regions. The introduction of the organic chromophore modifies the nature of the excited states from ^3^MLCT to ^3^LC centered on the naphthalimide chromophore [10,11,12].

Pursuing our work in this area, we sought to prepare another class of luminescent carbene complexes incorporating copper as inorganic chromophore and pyrene as organic chromophore. The high abundance of copper and its low cost compared to expensive third raw transition metals render this metal as an attractive choice to construct copper based luminescent materials.

Copper complexes display a small S_1_-T_1_ energy gap, ΔE_ST_ and thus enable efficient triplet harvesting by reverse intersystem crossing (RISC). Such a process is known as the aforementioned TADF mechanism [13,14]. The judicious placement of the pyrene luminophore via the carbene ligand in close proximity to the metal center provides access to a novel class of stable luminescent complexes with rich excited states [15].

**Figure 1:**
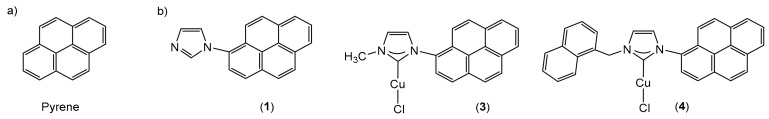
(**a**) Pyrene. (**b**) Imidazole-pyrenyl ligand (**1**) and the novel NHC–Cu(I) complexes (**3**) and (**4**) described in this work.

In this work, we describe the design of a novel class of luminescent NHC–copper (I) complexes containing a pyrene chromophore.(Figure 1) The effect of the alkyl substituent versus the naphthyl group on the carbene unit was investigated and showed that the latter increases the emissive properties of the compounds. 

Interestingly, all compounds showed comparable quantum yields to that of the parent pyrene molecule; remarkably, the complex with naphthyl-group increases the quantum yields by almost two-folds. Moreover, our complexes are strongly emissive when compared to classical *N*-heterocyclic coinage halogen complexes, which are often weakly emissive [16,17,18,19].

## 2. Results and Discussion

### 2.1. Synthesis and Characterization

**Scheme 1:**
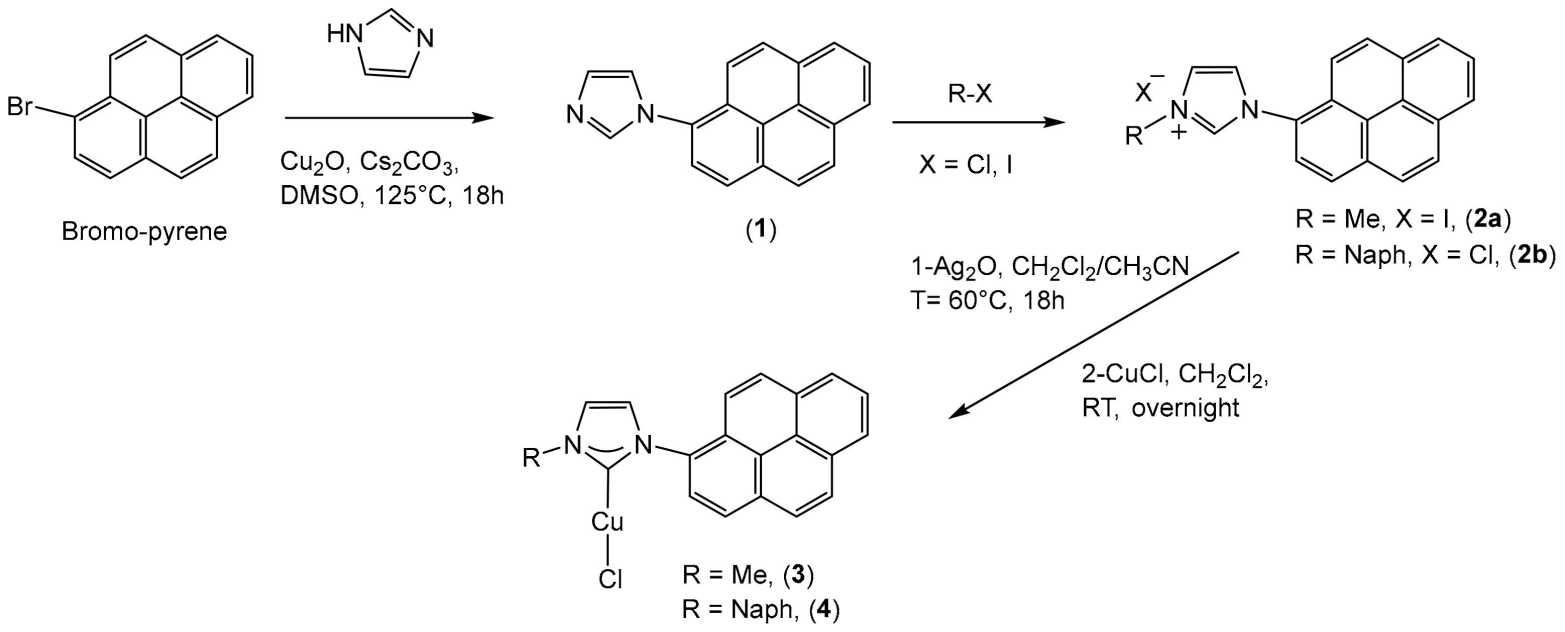
Synthesis of the imidazole-pyrene ligand and the related prenyl-NHC-Cu(I)-Cl (**3–4)** complexes containing pyrene luminophore.

Complexes **3–4** were obtained in three steps, according to a synthetic procedure developed by our group. The first step consists of the preparation of imidazole-pyrenyl ligand **1** from the commercially available Br-pyrene as starting material, which is *N*-arylated upon treatment with imidazole under heating in the presence of Cs_2_CO_3_ and a catalytic amount of Cu_2_O. Reaction workup, followed by column chromatography, provided analytically pure **1** in 92% yield. The second step consists in alkylation of **1** with the appropriate alkyl halide in THF to give the corresponding azolium salts (**2a**, **2b**) as pure white salts in 97% and 87% yields, respectively. Finally, the target pyrenyl-NHC-Cu(I)-Cl complexes (**3**, **4**) were obtained in 83% and 86% yields, respectively, by treatment of the azolium salts with CuCl using Ag_2_O via the transfer reagent method. (Scheme 1).

The ^1^H-NMR spectra of **1** recorded in CDCl_3_ confirm the formation of our imidazole-pyrenyl chromophore. Ligand **1** displays a multiple pattern for the aromatic protons of the pyrenyl-unit. The protons ascribed to the imidazolyl unit appear as two large singlets at δ 7.44 ppm and δ 7.83 ppm and show by integration 2H and 1H, respectively. The formation of the imidazolium salts **2a** and **2b**, is inferred by the appearance of the NC*H*N proton, which occurs at δ 9.77 and δ 10.15 ppm, respectively (see also Figures S1–S10). We also note that the crystal structure of pyrenyl-azolium salt containing a BF_4_^−^ as a counter anion was reported previously. However, the preparation follows an electrosynthetic procedure, which is completely different from the synthetic procedure, described in this work [20]. The pyrenyl-NHC-Cu-Cl complexes **3** and **4** were found to be stable in solution to allow full characterization. For instance, the ^13^C NMR of the carbenic carbon for these complexes display signals at around δ 179 ppm after prolonged acquisition. These compounds were kept under argon in the solid-state for several weeks without decomposition. 

Moreover, the identity of all molecules was ascertained by electrospray spectrometry. Complete spectroscopic characterizations (^1^H, ^13^C, MS) are given in the experimental section. Furthermore, the molecular structures of **3** and **4** were confirmed by single crystal X-ray diffraction study (vide infra).

### 2.2. X-ray Molecular Structures of (Pyrenyl-NHC-R)-Cu-Cl, (R= Me, 3; R = Naph, 4)

**Figure 2:**
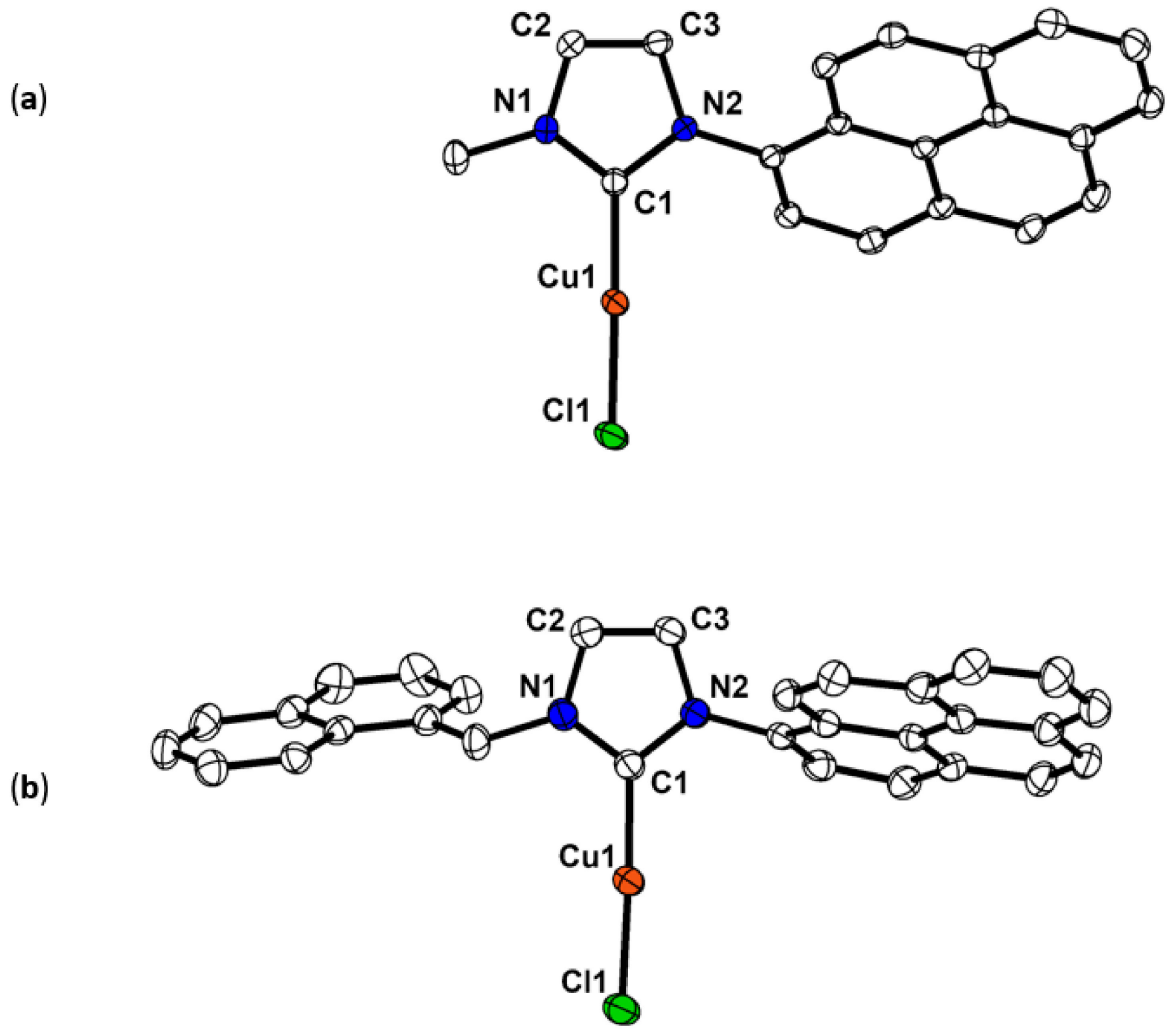
Crystal structures of **3** (**a**) and **4** (**b**). Thermal ellipsoids were drawn at 30% probability. Hydrogens were omitted for clarity. Selected bond distances (Å) and angles (°) for **3**: Cu1-Cl1 2.095(1), Cu1-C1 1.876(2), C1-N1 1.346(3), C1-N2 1.367(3), N1-C2 1.377(3), N2-C3 1.393(3), C2-C3 1.336(4), Cl1-Cu1-C1 176.44(8), Cu1-C1-N1 128.2(2) and Cu1-C1-N2 127.6(2). Selected bond distances (Å) and angles (°) for **4**: Cu1-Cl1 2.101(2), Cu1-C1 1.881(4), C1-N1 1.350(5), C1-N2 1.357(5), N1-C2 1.379(6), N2-C3 1.382(5), C2-C3 1.333(6), Cl1-Cu1-C1 176.6(2), Cu1-C1-N1 127.0(3), Cu1-C1-N2 128.8(3).

Suitable crystals of **3** and **4** were obtained by slow diffusion of cyclohexane into a solution of the complex in dichloromethane. The crystal structures were solved by X-ray diffraction and confirmed the formation and molecular structures of the target compounds (Figure 2a,b).

Both structures show common features. For instance, the NHC-Cu-Cl moiety is indeed attached at the 1-position of the pyrene luminophore. The two-coordinate copper complexes display linear configurations around the metal center with angles of 176.44(8)° for **3** and C1 176.6(2)° for **4**, in line with those obtained for related two-coordinated NHC-Cu-Cl complexes [21,22,23]. The carbene-Cu bond distances are 1.876(2) Å and 1.881(4) Å for **3** and **4,** respectively, and the Cu-Cl bond distances are of 2.095(1) Å and 2.101(2) Å, respectively. These bond distances are similar to those found in related two-coordinate NHC-Cu(I) chloride compounds [23,24].

**Figure 3:**
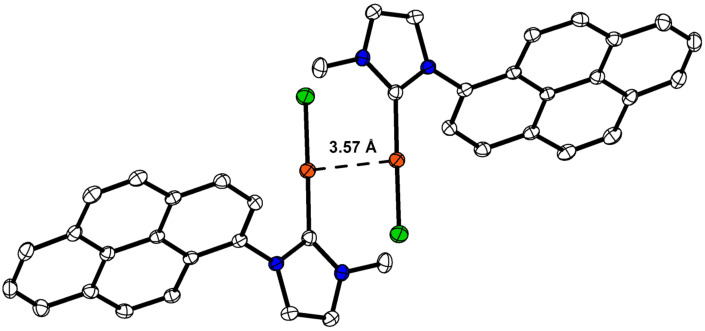
Packing of **3** showing weak Cu---Cu contact between two adjacent molecules at the supramolecular level.

Examining the packing of the molecules in the crystal of **3** revealed the presence of weak Cu(I)---Cu(I) contacts between two adjacent molecules at *d* = 3.573(1) Å (Figure 3) [25]. As for complex **4**, the packing shows a distance between the two Cu(I) centers at d = 3.797(1) Å, which precludes the presence of cuprophilic interactions [26]. This difference might be the consequence of the large naphthyl-group compared to the CH_3_-group on the carbene unit, which generates more steric hindrance and eventually moves the molecules further away suppressing metal---metal interaction at the supramolecular level. Further weak secondary interactions occurring in the packing of **3** are an intermolecular C-H…Cl hydrogen bonding of 2.762 Å between H22 of the pyrene cycle and the chlorido ligand of a neighbored molecule, thus generating a kind of 1D supramolecular ribbon.

For complex **4**, the chlorido ligand even triply interacts with H10 (2.828 Å), H17 (2.902 Å), and H22 (2.762 Å) of the aryl cycles. 

### 2.3. Absorption Properties

**Figure 4:**
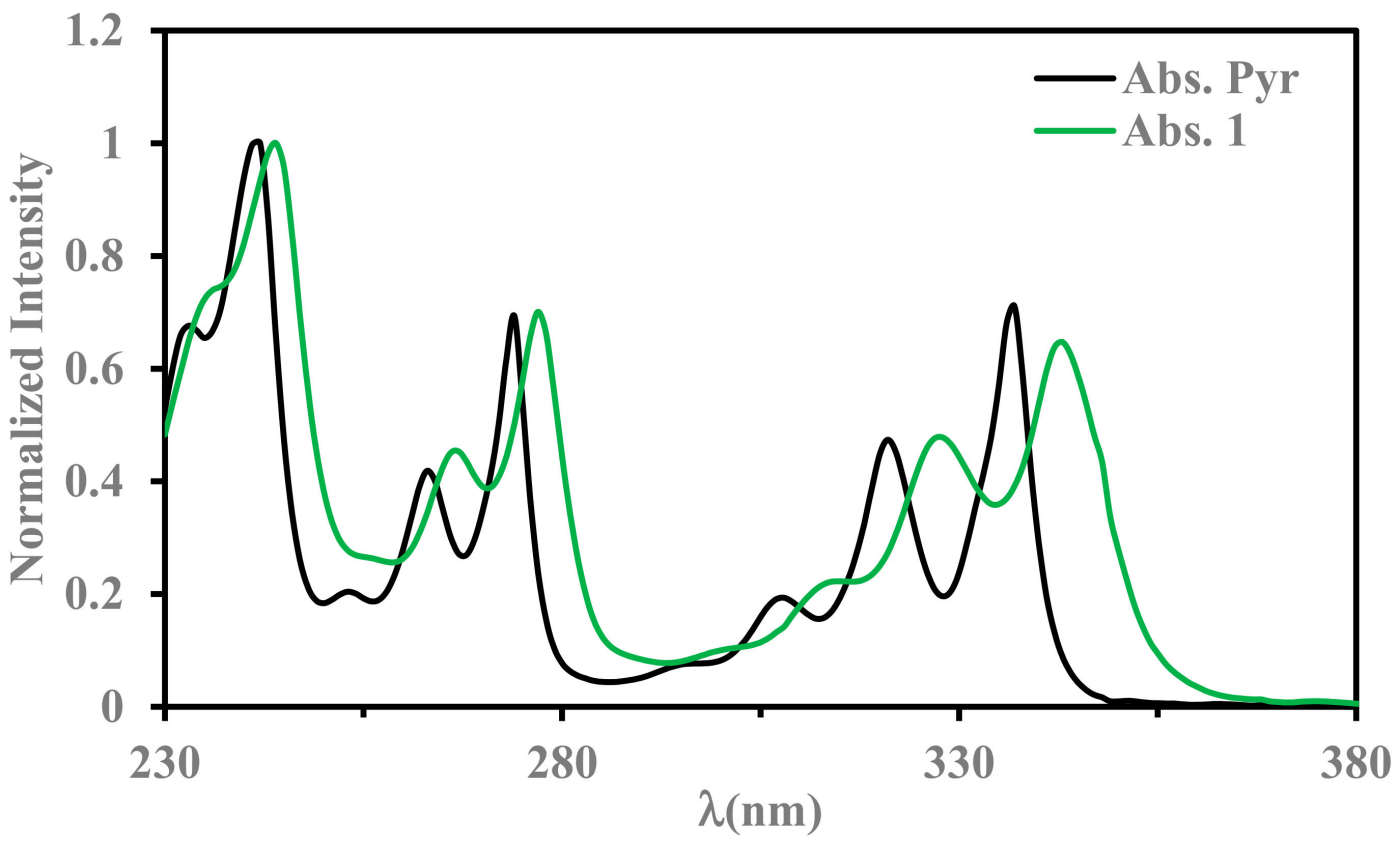
Absorption spectra of pyrene (Pyr) and **1** in CH_2_Cl_2_ solution at rt.

The UV absorption profiles of ligand **1** and complexes **3–4** in dilute CH_2_Cl_2_ solution at room temperature are shown in Figure 4, and pertinent absorption data are presented in Table 1.

The ligand **1** exhibits vibronically resolved intense absorption bands with λ_max_ = 343 nm assigned to ^1^π-π* transitions centered at the pyrene chromophore [27]. The presence of the imidazole moiety at the C1-position induces a bathochromic shift of all bands by about 2–6 nm indicating a lowering of the energy difference between the HOMO-LUMO orbitals when compared to the free pyrene (Figure 4). This might indicate that attachment of the imidazole to the pyrene extends the π-conjugation in ligand **1**.

**Figure 5:**
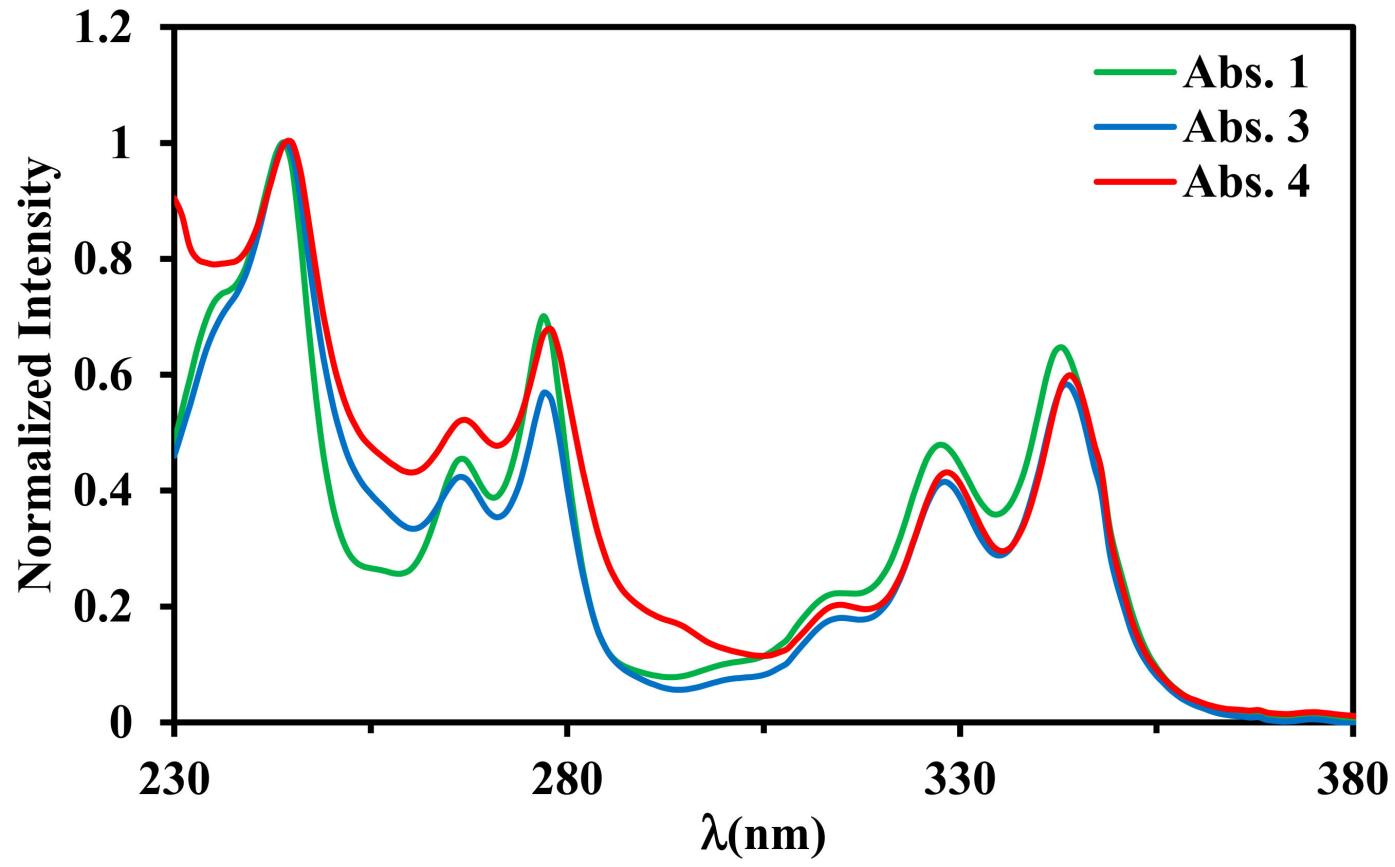
Absorption spectra of ligand **1** and complexes **3–4** in CH_2_Cl_2_ solution at rt.

The Cu(I) complexes **3–4** present similar vibronic absorption bands to that of **1** in the UV region with λ_max_ = 344 nm for **3** and for **4** as well (Figure 5). These absorption bands are again associated with the spin-allowed π-π* ligand-centered (LC) transitions from the pyrene ligand [27].

### 2.4. Luminescence Properties

**Figure 6:**
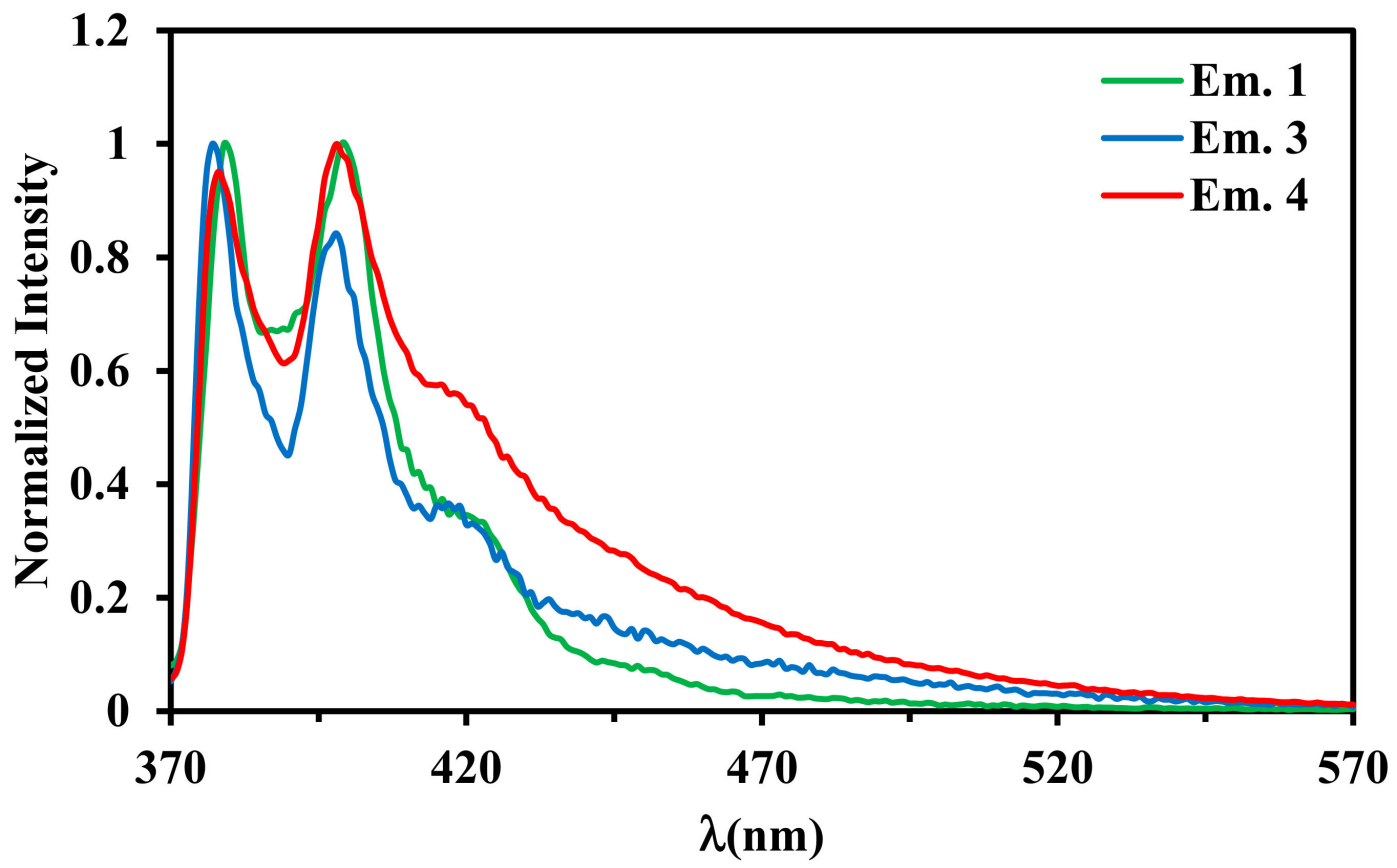
Normalized emission spectra of ligand **1** and carbene complexes **3–4** in de-aerated CH_2_Cl_2_ solution at rt.

**Figure 7:**
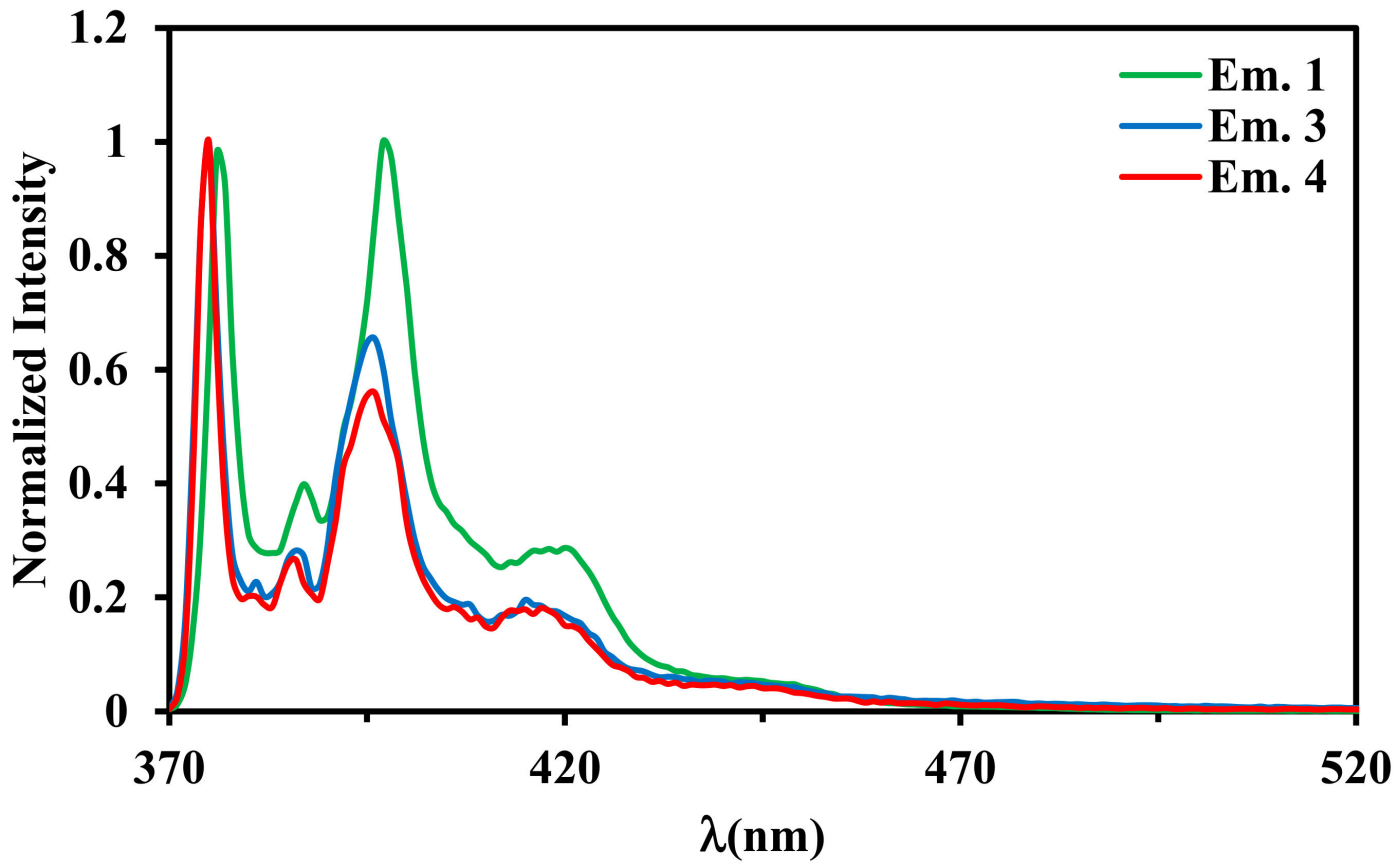
Normalized emission spectra of ligand **1** and complexes **3–4** in CH_3_OH:C_2_H_5_OH (1:4) glassy solution at 77 K.

The photoluminescence properties of ligand **1** and the complexes **3–4** were investigated in CH_2_Cl_2_ solution at room temperature (298 K) and in CH_3_OH: C_2_H_5_OH (1:4) glassy solution at 77 K. The normalized emission spectra of the ligand and complexes at 298 K are shown in Figure 6, and the spectra at 77 K are given in Figure 7. The emission maxima (λ_max_), photoluminescence quantum yields (ϕ) and lifetimes (τ) are collected in Table 2.

The ligand **1** exhibits at room temperature a relatively broad and structured emission bands, with λ_max_ at 379 nm (Figure 5) with quantum yield (ϕ = 39 %), and short lifetimes in the nanosecond regime τ = 32.13 ns. This behavior can be assigned to the fluorescence of the pyrene unit of the molecule. For comparison purposes, the parent pyrene molecule under similar experimental condition displays a lower quantum yield in CH_2_Cl_2_ (ϕ = 27 %) [28].

The emission spectrum of **1** in a CH_3_OH:C_2_H_5_OH glassy solution at 77 K is reported in Figure 7. In comparison with its spectrum recorded at rt, we notice the presence of a similar pattern but with sharper bands.

The Cu(I) carbene complexes **3–4**, display in CH_2_Cl_2_ solution at room temperature, similar broad and structured emission bands in comparison to the free ligand **1** (Figure 6), with λ_max_ at 377 nm for both complexes, with lifetimes τ = 8.72 ns and τ = 7.79 ns, respectively, indicating that the observed emissions are fluorescent in nature. The displayed vibronic structure indicates a predominantly ^1^LC π-π* character of the emissive excited state centered at the pyrenyl chromophore. This is in line with the photoluminescence behavior of other metalated complexes containing pyrene motifs [17]. The emission spectra of the Cu(I) carbene complexes **3** and **4** at room temperature also show a longer tail that extend into the visible region when compared to ligand **1**.

It is worth mentioning that the photoluminescence quantum yields of our complexes **3**, **4** are (ϕ = 28 %) and (ϕ = 42 %), (Table 2) which are higher than that observed for the pyrene parent molecule suggesting that the presence of the “NHC-Cu-Cl” unit provides extra rigidity to the system and hence increases the emission efficiency. Moreover, it should be mentioned that mononuclear-NHC-halogenated complexes have been investigated in the literature and are often described as weakly or even non-emissive [16,17,19,29].

**Figure 8:**
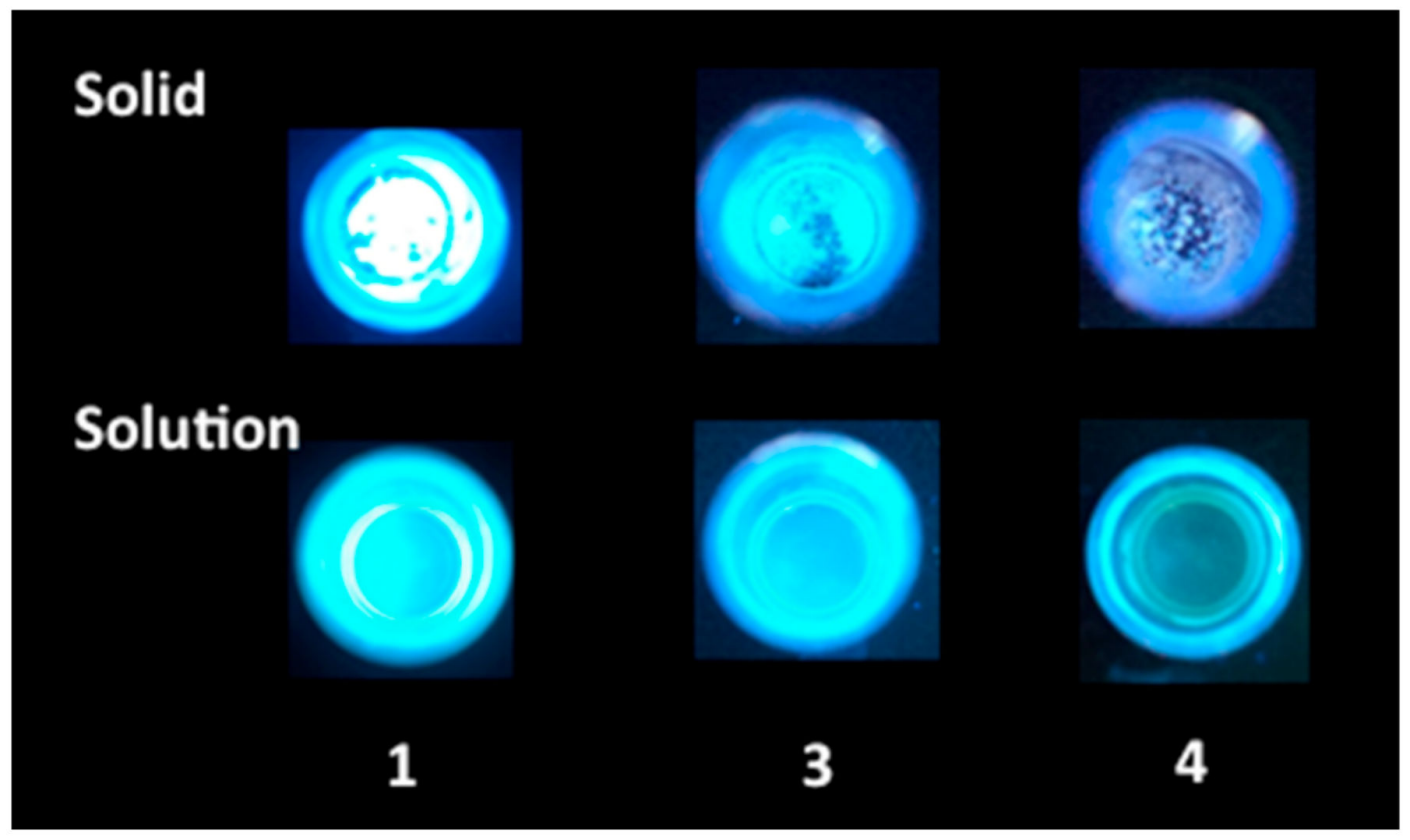
Photograph of ligand **1** and complexes **3**, **4** upon irradiation with a UV lamp in the dark at 365 nm.

The photograph of ligand **1** and carbene complexes **3**, **4** in CH_2_Cl_2_ and in solid-state upon irradiation with a UV lamp in the dark at 365 nm is given in Figure 8.

At low temperature in CH_3_OH:C_2_H_5_OH glassy solution at 77 K, complexes **3** and **4** were found to be luminescent (Figure 7). The spectra of the two complexes **3** and **4** show sharper bands at 77 K when compared to those recorded at rt, and they are slightly blue shifted relative to the rt emissions. No phosphorescence was detected at 77 K.

The solid-state emission spectra of ligand **1** and complexes **3–4** recorded at room temperature displayed broad structureless bands for all compounds with λ_max_ at 470 nm, 457 nm and 462 nm and lifetimes of τ = 27.23 ns and τ = 1.13 ns and τ = 7.79 ns, respectively (Figure S11). This type of broad emission can be tentatively assigned to the excimers formed between pyrene molecules in the solid-state [30,31]. The spectra of **1** and complexes **3–4** were also recorded at 77 K and provided a similar large band pattern.

## 3. Conclusions

In this paper, we have described the synthesis of a novel fluorescent imidazole-pyrenyl ligand **1**, which allowed the preparation of some stable *N*-heterocyclic carbene chloride copper (I) complexes (**3–4**) containing a pyrenyl chromophore. The X-ray molecular structures of both complexes are described and confirm the identity of the target molecules. These complexes represent the first examples of such compounds and pave the way to the placement of other inorganic metal chromophores to the pyrene fluorophore. At room temperature, ligand **1** and the Cu(I) complexes **3–4** display blue fluorescence at 379 nm and 377 nm originating mainly from the pyrene ligand. All of our molecules were found to be strongly emissive. Remarkably, the quantum yields of **1**, **3** and **4** in solution were found to be higher than that of the pyrene parent molecule under same experimental conditions. These results highlight the importance of the NHC-Cu-Cl moiety, which brings extra rigidity to the system and enhances its emission properties and this should be also compared to the known mononuclear-NHC-halogenated complexes, which are often weakly or even non-emissive.

## 4. Materials and Methods

Apart from the chlorides/anions metathesis workup, which has been performed under air, all other experimental manipulations were carried out under argon atmosphere using Schlenk tube techniques. Standard techniques were used for the solvents purification. Deuterated solvents and commercially available reagents were used as received unless otherwise specified. A Bruker Avance 300 NMR or Avance NEO 400 spectrometer was used for recording the ^1^H NMR and ^13^C NMR spectra in CD_2_Cl_2_ or CDCl_3_. For the UV-Vis spectra, a VARIAN-Cary 300 array spectrophotometer was used. Chemical shifts are reported in ppm downfield from tetramethylsilane and are referenced to the residual hydrogen signal of deuterated solvents (CHD_2_NO_2_ at 4.33 ppm, CHDCl_2_ at 5.32 ppm) and the residual solvent carbon signal (CD_3_NO_2_ at 61.4 ppm, CD_2_Cl_2_ at 53.5 ppm) for ^13^C NMR. A Jobin-Yvon Fluoro Log 3 spectrofluorometer equipped with a R928P detector was employed for recording the steady state excitation and emission spectra both in the solid-state (298 K and 77 K) and in solution at room temperature.

**Synthesis of 1-(pyren-1-yl)-1*H*-imidazole (1)**

Under argon atmosphere, 1-bromo-pyrene (1.5 g, 1.0 equiv.), imidazole (546 mg, 1.5 equiv.), Cs_2_CO_3_ (3.45 g, 2.0 equiv.), 2,2,6,6-tetramethyl-3,5-heptanedione(DPM) (197 mg, 0.2 equiv), and Cu_2_O (153 mg, 0.2 equiv.) were introduced into a Schlenk tube equipped with magnetic stirrer. Then DMSO (6 mL) was added with vigorous stirring at room temperature. The reaction mixture was sealed and heated at 100 °C, for 18 h. Then mixture was cooled to room temperature and water (30 mL) was added and extracted with ethyl acetate. The organic phase was separated and dried with anhydrous MgSO4. Subsequently, the product was purified using column chromatography to obtain a white solid (1.317 g, 92% yield). ^1^H NMR (400 MHz, CDCl_3_) δ 8.32–8.20 (m, 3H, H_py_), 8.21–8.05 (m, 4H, H_py_), 7.95 (dd, J = 8.0, 2.4 Hz, 2H, H_py_), 7.83 (d, J = 9.2 Hz, 1H, H_im_), 7.44 (s, 2H, H_im_). ^13^C NMR (400 MHz, CDCl_3_) δ 131.51, 131.18, 130.88, 130.72, 129.40, 128.52, 126.98, 126.75, 126.72, 126.19, 125.85, 125.07, 124.79, 124.25, 123.87, 121.05, 77.37, 77.26, 77.06, 76.74. HRMS (ESI) *m*/*z*: [M+H]^+^ Calcd for C_19_H_12_N_2_H 269.1073. Found 269.106.

**Synthesis of 3-methyl-1-(pyren-1-yl)-1*H*-imidazole-3-ium iodine (2a).**

1-(pyren-1-yl)-1*H*-imidazole (800 mg, 1.0 equiv) was introduced into a round bottom flask equipped with magnet stirrer and a rubber stopper. THF (8 mL) was added to the reaction mixture, then MeI (3.6 mL, 2.0 equiv), was added dropwise and the system was sealed. The reaction mixture was heated at 60°C for 48 h. Addition of Et_2_O (20 mL) to the reaction mixture, provided a white precipitate which was filtered off and washed with Et_2_O (2 × 5 mL), and then dried under vacuum. (1.189 g, 97% yield). ^1^H NMR (400 MHz, DMSO-*d_6_*) δ 9.77 (s, 1H, H_im_), 8.54 (d, J = 8.2 Hz, 1H, H_im_), 8.49 (dd, J = 7.6, 3.1 Hz, 1H, H_py_), 8.46–8.37 (m, 2H, H_py_), 8.39–8.28 (m, 3H, H_py_), 8.23 (t, J = 7.7 Hz, 1H, H_py_), 8.15 (t, J = 1.8 Hz, 1H, H_py_), 7.93 (d, J = 9.2 Hz, 1H, H_im_), 4.10 (s, 3H, H_Me_). ^13^C NMR (400 MHz, DMSO-*d_6_*) δ 139.17, 132.64, 131.05, 130.63, 130.53, 129.75, 128.49, 127.81, 127.45, 127.39, 126.97, 126.13, 125.61, 125.36, 124.93, 124.85, 124.68, 124.36, 123.53, 120.73, 36.86. HRMS (ESI) *m*/*z*: [M]^+^ Calcd for C_20_H_15_N_2_ 283.1226. Found 283.1230.

**Synthesis of 3-(naphthalen-1-ylmethyl)-1-(pyren-1-yl)-1*H*-imidazole-3-ium chloride (2b).**

A round bottom flask equipped with a stirrer bar was charged with 1-(pyren-1-yl)-1*H*-imidazole (1.0 g, 1.0 equiv) and 1-(chloromethyl)naphthalene (985 mg, 1.5 equiv). Dioxane (20 mL) was added and the reaction mixture was heated at 100 °C for 48 h in a sealed system, during which a white precipitate was formed. The solid was filtered and washed with dioxane (2 × 10 mL), then with Et_2_O (2 × 10 mL), and dried under vacuum to give a white microcrystalline solid (1.450 g, 87% yield). ^1^H NMR (400 MHz, DMSO-*d_6_*) δ 10.15 (t, J = 1.6 Hz, 1H, H_im_), 8.57–8.45 (m, 3H, H_py_), 8.45–8.32 (m, 6H, H_py_), 8.28–8.19 (m, 2H, H_naph_), 8.09 (d, J = 8.2 Hz, 2H, H_im_), 7.84–7.61 (m, 4H, H_naph_), 6.20 (s, 2H, H_CH2_). ^13^C NMR (400 MHz, DMSO-*d_6_*) δ 139.15, 133.97, 132.68, 131.06, 131.02, 130.70, 130.51, 130.30, 130.26, 129.78, 129.45, 128.54, 128.45, 127.84, 127.76, 127.48, 127.00, 126.97, 126.24, 126.07, 125.96, 125.59, 125.05, 124.34, 123.83, 123.54, 123.50, 120.47, 66.81. HRMS (ESI) *m*/*z*: [M]^+^ Calcd for C_30_H_21_N_2_ 409.1699. Found 409.1699.

**General procedure for the preparation of Cu(I) *N*-heterocyclic carbene complexes (3, 4).**

The azolium salt either **2a** or **2b** was introduced into a dry Schlenk tube kept under argon. Then degassed dried solvents CH_2_Cl_2_ and CH_3_CN (1V:1V = 8 mL/8 mL) were introduced with Ag_2_O (2.0 eq.). The solution was refluxed at 60 °C for overnight under light protection using aluminum foil. The mixture was cooled and an appropriate amount of charcoal was introduced and the reaction mixture was filtered to another Schlenk tube containing CuCl (2.0 eq.). CH_2_Cl_2_ (16 mL) was added and the reaction mixture was heated for overnight at 60° C. Then, CuCl (1 eq) was introduced and the reaction was left to stir for 24 h. The mixture was cooled then filtered through celite in order to remove a grey precipitate and washed with CH_2_Cl_2_. The crude product was extracted by CH_2_Cl_2_. The solvent was removed under vacuum and the precipitate was recrystallized from CH_2_Cl_2_/cyclohexane to give microcrystalline solids, which was filtered and dried, under vacuum.

Compound **3** was obtained as off-white microcrystalline solid (144 mg, 83%) using (200 mg, 0.4878 mmol) of **2a**. ^1^H NMR (400 MHz, CD_2_Cl_2_) δ 8.41–8.30 (m, 3H, H_py_), 8.31–8.19 (m, 3H, H_py_), 8.20–8.10 (m, 2H, H_py_), 7.85 (d, J = 9.2 Hz, 1H, H_py_), 7.42 (s, 1Hm, H_im_), 7.30 (s, 1H, H_im_), 4.10 (s, 3H, H_Me_). ^13^C NMR (400 MHz, CD_2_Cl_2_) δ 178.95 (C-Carbenic), 131.97, 131.20, 130.67, 129.39, 128.65, 127.06, 126.76, 126.34, 125.96, 125.03, 124.90, 124.72, 124.15, 120.77, 38.6 (CH_3_-). HRMS (ESI) *m*/*z*: [M]^+^ Calcd for (C_20_H_14_N_2_)_2_Cu 627.1604. Found 627.159.

Compound **4** was also obtained as white microcrystalline solid using (200 mg, 0.4494 mmol) of **2b** (196 mg, 86%). ^1^H NMR (400 MHz, CD_2_Cl_2_) δ 8.34 (dd, J = 13.5, 7.7 Hz, 3H, H_naph_), 8.29–8.10 (m, 6H, H_py_), 8.02 (d, J = 6.4 Hz, 2H, H_naph_), 7.83 (d, J = 9.2 Hz, 1H, H_naph_), 7.75–7.59 (m, 4H, H_naph_), 7.36 (s, 1H, H_im_), 7.14 (s, 1H, H_im_), 6.02 (s, 2H, H_CH2_). ^13^C NMR (400 MHz, CD_2_Cl_2_) δ 179.03 (C-Carbenic), 134.05, 132.04, 131.20, 131.12, 130.84, 130.66, 129.80, 129.48, 129.03, 128.69, 127.85, 127.18, 127.05, 126.78, 126.39, 126.00, 125.50, 125.04, 124.91, 124.73, 124.14, 123.08, 120.68, 65.65, 15.08. HRMS (APCI) *m*/*z*: [M]^+^ Calcd for C_30_H_20_CuN_2_ 471.0917. Found 471.0927.

**X-ray crystal structure determination.** Single crystals were selected, mounted and transferred into a cold nitrogen gas stream. Intensity data was collected with a Bruker Kappa APEX II system using micro-source Cu-Kα radiation. Unit cell parameters determination, data collection strategy, integration and absorption correction were carried out with the Bruker APEX3 suite of programs. The structures were solved with SHELXT and refined anisotropically by full-matrix least-squares methods with SHELXL using WinGX. The structures were deposited at the Cambridge Crystallographic Data Centre with numbers CCDC 2249001 and CCDC 2249002 and can be obtained free of charge via www.ccdc.cam.ac.uk.

**Crystal data for 3.** C_20_H_14_ClCuN_2_, monoclinic P 2_1_/c, a = 13.1563(5) Å, b = 16.1523(6) Å, c = 7.7591(3) Å, α = γ = 90°, β = 102.650(2)°, V = 1608.82(11) Å^3^, Z = 4, colorless needle 0.25 × 0.05 × 0.03 mm^3^, μ = 3.447 mm^−1^, min/max transmission = 0.67/0.96, T= 200(1) K, λ = 1.54178 Å, θ range = 4.40° to 66.58°, 10,915 reflections measured, 2833 independent, R_int_ = 0.0318, completeness = 0.995, 217 parameters, 0 restraints, final R indices R1 [I > 2σ(I)] = 0.0348 and wR2 (all data) = 0.0961, GOF on F^2^ = 1.024, largest difference peak/hole = 0.42/−0.36 e·Å^−3^.

**Crystal data for 4.** C_30_H_20_ClCuN_2_, monoclinic P 2_1_/n, a = 7.6040(6) Å, b = 23.8164(19) Å, c = 14.3051(13) Å, α = γ = 90°, β = 94.615(5)°, V = 2582.3(4) Å^3^, Z = 4, colorless needle 0.76 × 0.04 × 0.03 mm^3^, μ = 2.287 mm^−1^, min/max transmission = 0.50/1.00, T= 200(1) K, λ = 1.54178 Å, θ range = 3.62° to 66.58°, 17,321 reflections measured, 4540 independent, R_int_ = 0.1090, completeness = 0.998, 307 parameters, 0 restraints, final R indices R1 [I > 2σ(I)] = 0.0563 and wR2 (all data) = 0.1481, GOF on F^2^ = 0.957, largest difference peak/hole = 0.33/−0.54 e·Å^−3^.

**Photophysical measurements**. Steady-state excitation and emission spectra in the solid-state (298 K and 77 K) and in solution at room temperature were recorded on a Jobin-Yvon Fluoro Log 3 spectrofluorometer equipped with a R928P detector. Luminescence quantum yields Ø_L_ of complexes at 298 K were determined using pyrene (Ø_L_ = 27%) as a luminescence quantum yield standard because its fluorescence emission is in the same range as our samples [28]. Solid-state photophysical studies were carried out with solid samples contained in a quartz tube inside a quartz-walled Dewar flask. Measurements of the solid-state sample at 77 K were similarly conducted with liquid nitrogen filled in the optical Dewar flask. All solutions for photophysical studies were prepared in dry dichloromethane spectrophotometric grade. Luminescence decay signals were recorded using a FL-R928P-TCSPC apparatus equipped with a deltadiode source. Analysis of luminescence decay profiles against time was accomplished using the Decay Analysis Software DAS6.

## Figures and Tables

**Table 1:** Absorption data.

	λ_max_, nm (ε × 10^−3^ M^−1^ cm^−1^) *^a^*
**1**	235 *sh* (45), 244 (62.2), 267 (28.4),277 (43.6), 313 (13.5), 327 (30.1), 343 (40.6), 374 (0.6)
**3**	236 *sh* (35), 244 (49.6), 267 (21.1), 277 (28.3), 314 (8.9), 328 (20.6), 344 (29.2), 375 (0.5)
**4**	245 (52.4), 268 (27.2), 278 (35.4), 314 (10.8), 328 (22.5), 344 (21.2), 375 (1.1)

*^a^* In CH_2_Cl_2_ solution at rt; *sh* is shoulder.

**Table 2:** Emission data.

	rt			77 K
	λ_max_, nm *^a^*	ϕ (%) *^a^*	τ, ns *^a^*	λ_max_, nm *^b^*
**1**	379	39	32.13	397
**3**	377	28	8.72	375
**4**	377	42	7.79	375

*^a^* In CH_2_Cl_2_ solution at rt. *^b^* In CH_3_OH:C_2_H_5_OH (1:4) glassy solution at 77 K.

## Supplementary Materials

The following supporting information can be downloaded at: https://www.mdpi.com/article/10.3390/molecules28104025/s1. Figures S1–S10: 1H and 13C NMR spectra of all novel compounds **1**, **2a**, **2b**, **3** and **4.** Normalized emission spectra of ligand **1** and complexes **3** and **4** in solid-state at room temperature (Figure S11). Emission decay (S12–S14) of compounds **1**, **3** and **4** in CH_2_Cl_2_ at room temperature. Emission decay (S15–S17) of compounds **1**, **3** and **4** in solid-state at room temperature.
