# Supplementary material for: N-Heterocyclic Carbene Copper (I) Complexes Incorporating Pyrene Chromophore: Synthesis, Crystal Structure, and Luminescent Properties
Source: Molecules. 2023 May 11;28(10):4025. doi: 10.3390/molecules28104025 (PMC10223749; doi:10.3390/molecules28104025)
Supplement: Supplementary file 1 [file molecules-28-04025-s001.zip › molecules-2360093-supplementary.pdf]

Supporting Information

N-Heterocyclic Carbene Copper (I) Complexes Incorporating Pyrene Chromophore: Synthesis, Crystal Structure, and Luminescent Properties

Yaping Cheng [1], Geoffrey Gontard [1], Abderrahim Khatyr [2], Michael Knorr [2] and Hani Amouri [1,*]

[1] Institut Parisien de Chimie Moléculaire (IPCM), UMR CNRS 8232, Sorbonne Université-Campus Pierre et Marie Curie, 4 Place Jussieu, CEDEX 05, 75252 Paris, France; yaping.cheng@sorbonne-universite.fr (Y.C.); geoffrey.gontard@sorbonne-universite.fr (G.G.)
[2] Institut UTINAM, UMR CNRS 6213, Université de Franche-Comté, 16 Route de Gray, 25030 Besançon, France; abderrahim.khatyr@univ-fcomte.fr (A.K.); michael.knorr@univ-fcomte.fr (M.K.)
* Correspondence: hani.amouri@sorbonne-universite.fr

TABLE OF CONTENTS

Figures S1–S10 (^{1}H, ^{13}C) -NMR spectra of compounds (**1**, **2a**, **2b**, **3** and **4**).
Figure S11 Normalized emission spectra of ligand **1** and complexes **3-4** in solid-state at rt.
Figures S12–S14 Emission decay of compounds **1, 3** and **4** in CH_2Cl_2 at rt.
Figures S15–S17 Emission decay of compounds **1, 3** and **4** in solid-state at rt.

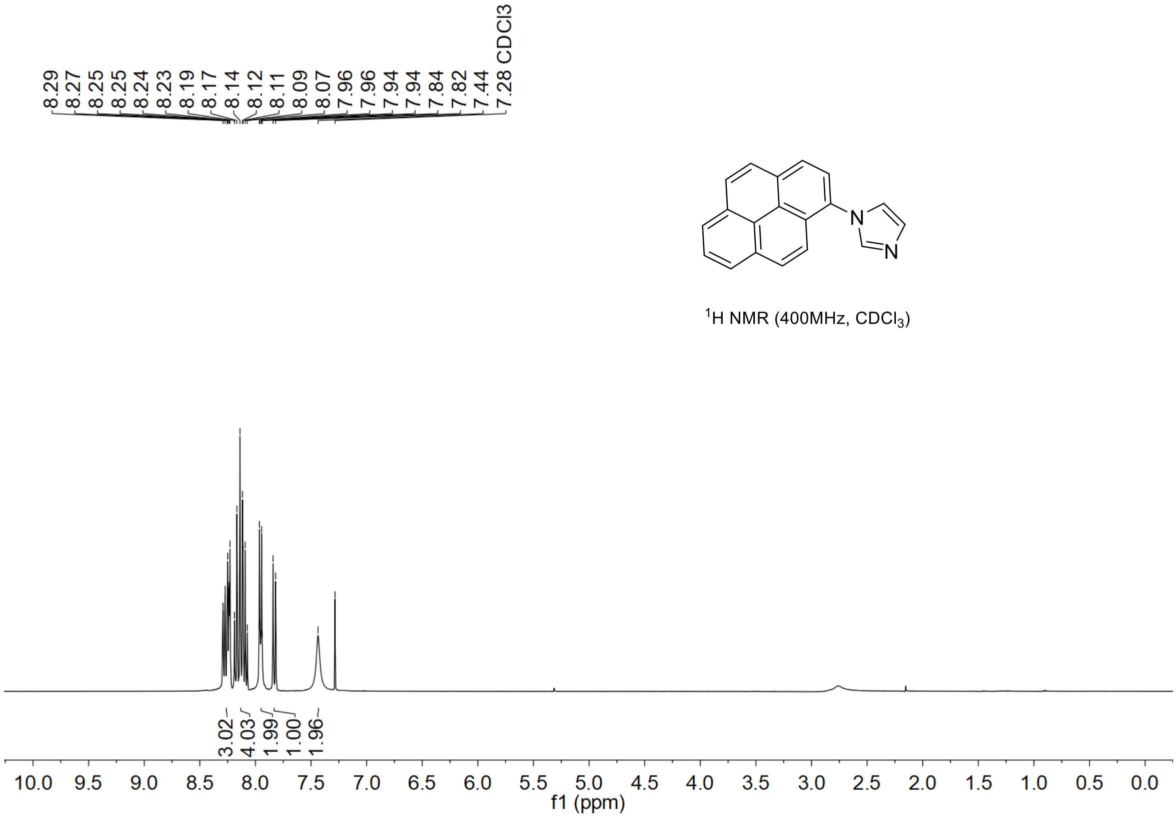

Figure S1. ^{1}H NMR of compound **1** in $CDCl_3$

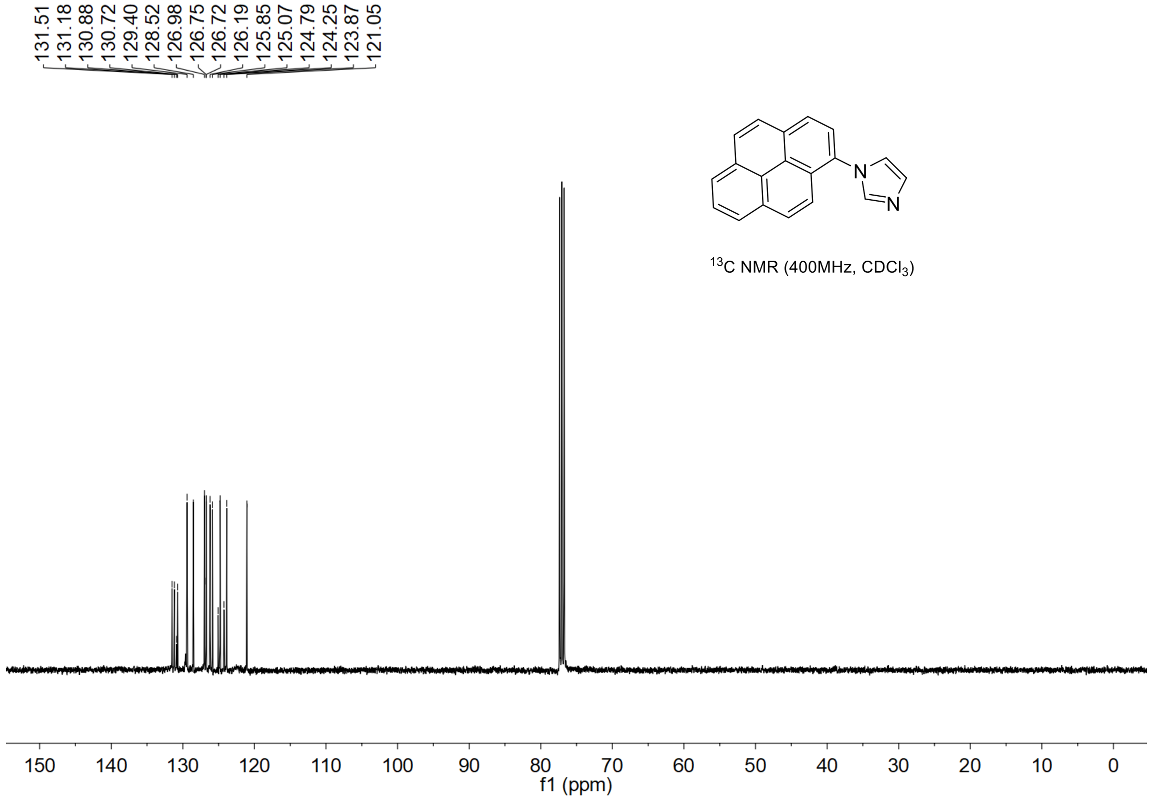

Figure S2. ^{13}C NMR of compound **1** in $CDCl_3$

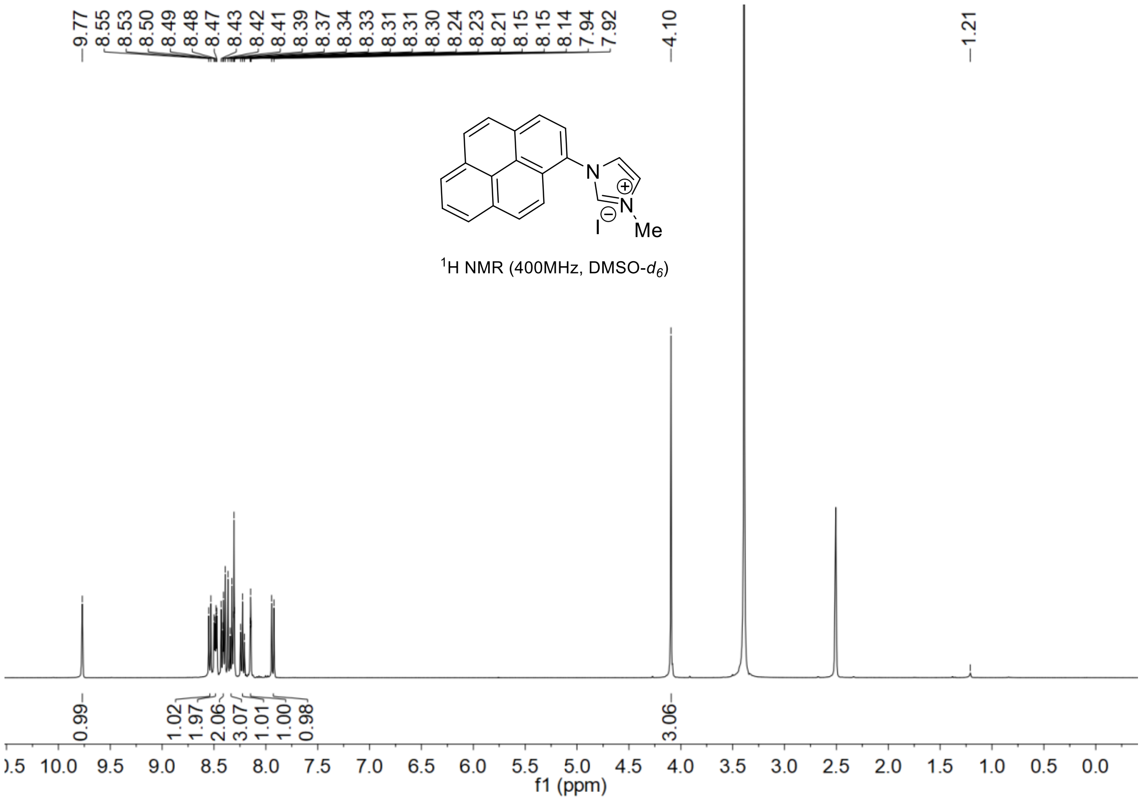

Figure S3. ^{1}H NMR of compound **2a** in DMSO

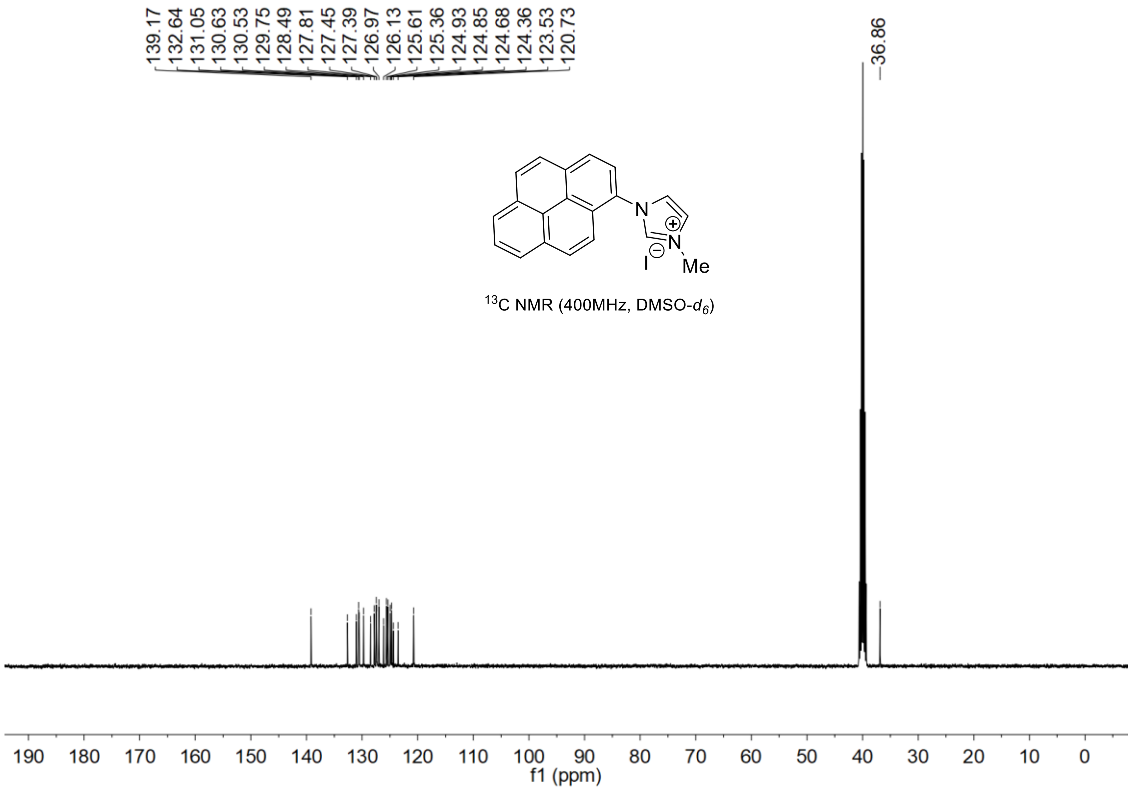

Figure S4. ^{13}C NMR of compound **2a** in DMSO

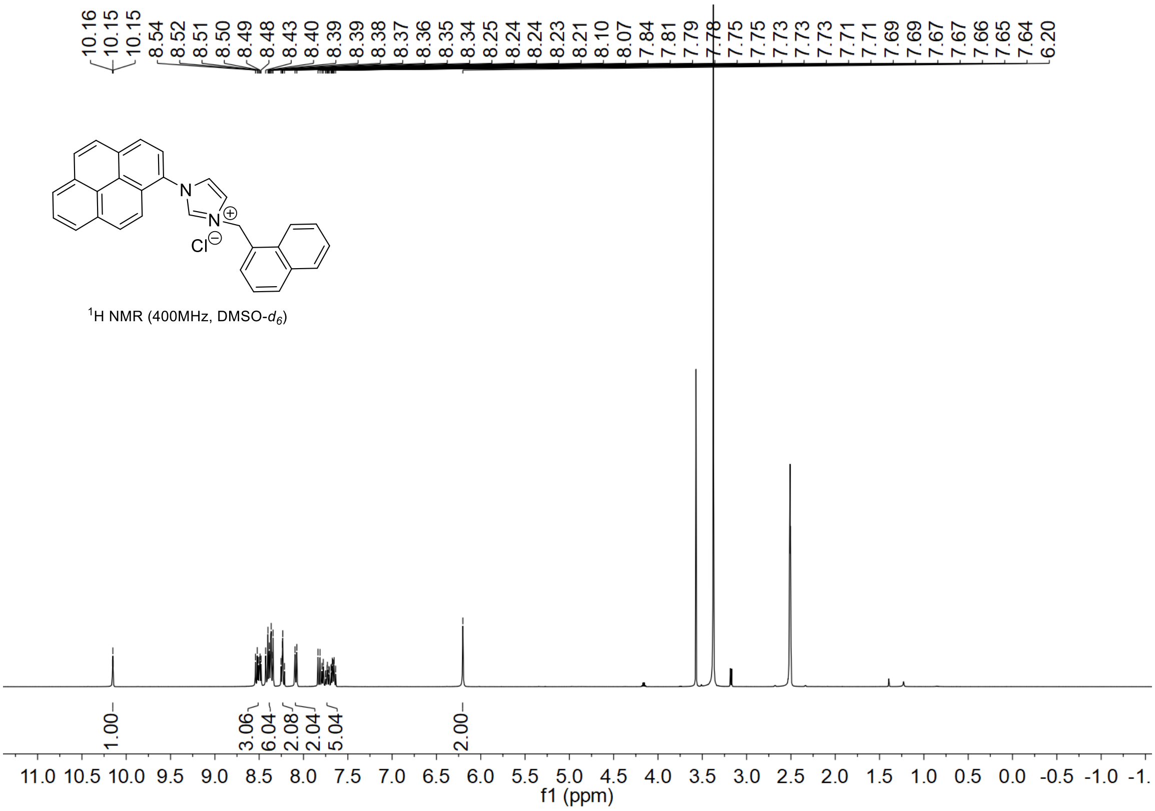

Figure S5. ^{1}H NMR of compound **2b** in DMSO

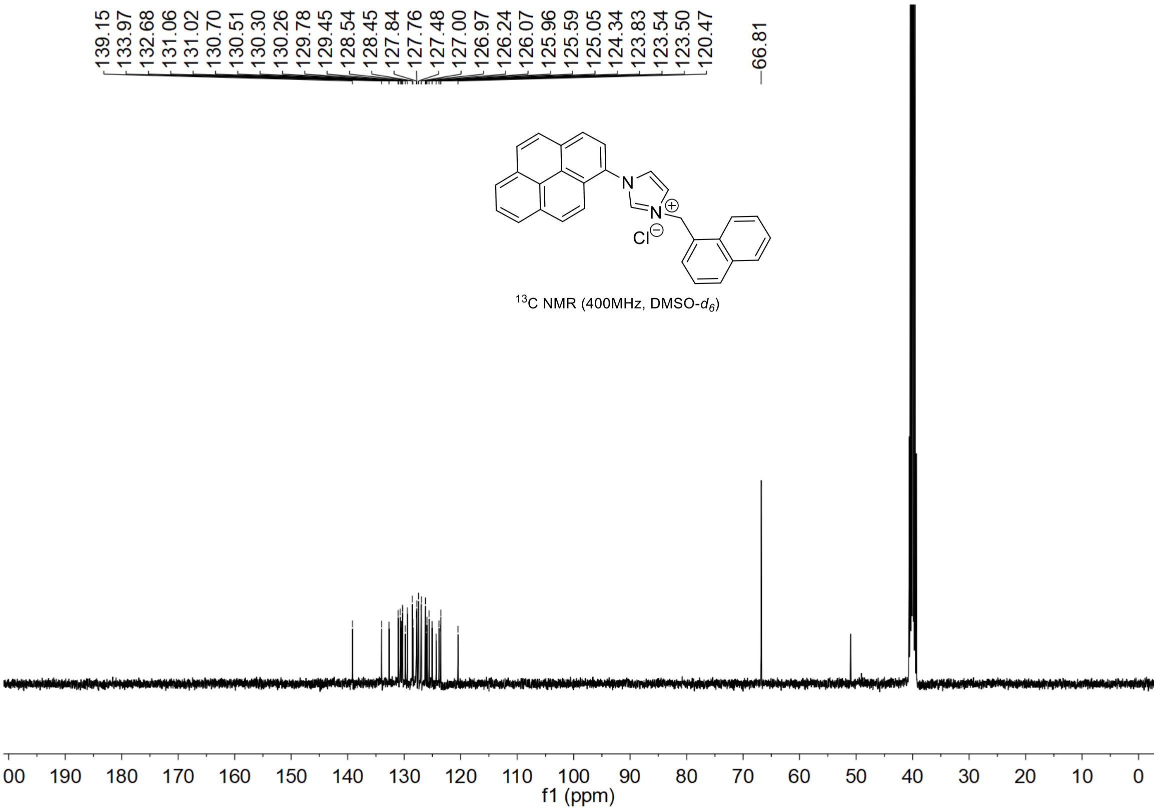

Figure S6. ^{13}C NMR of compound **2b** in DMSO

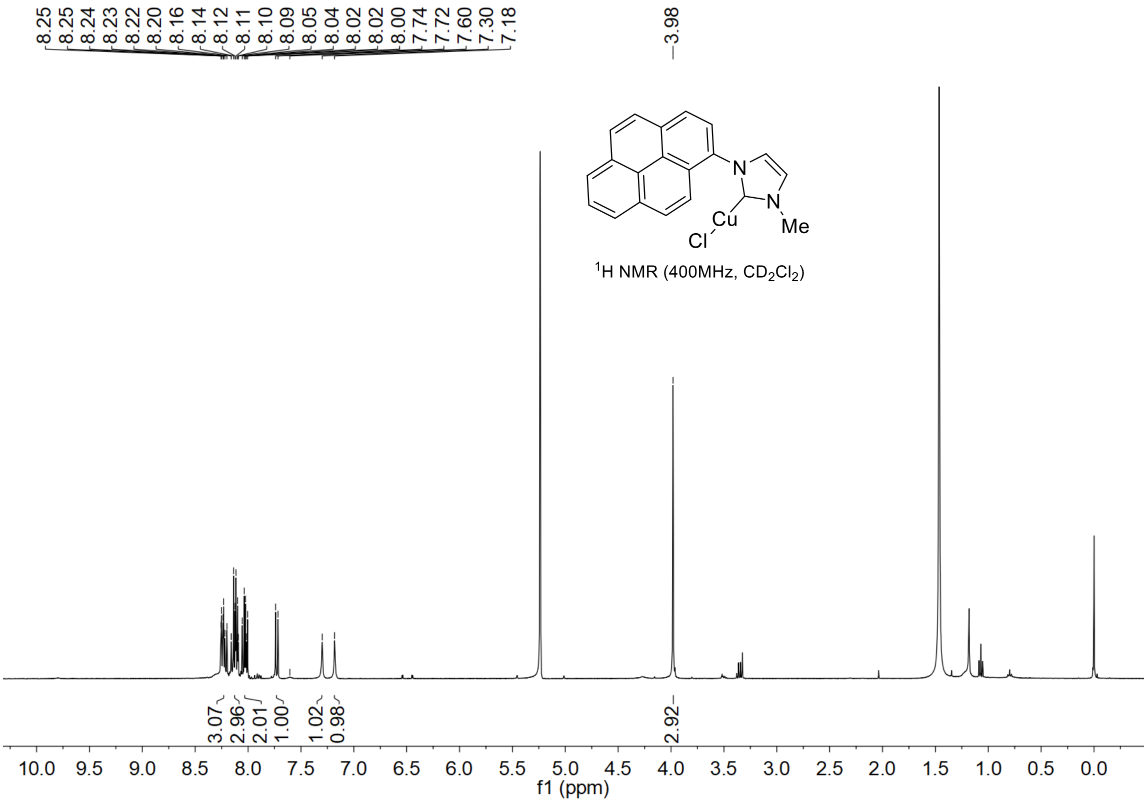

Figure S7. ^{1}H NMR of complex **3** in CD_2Cl_2

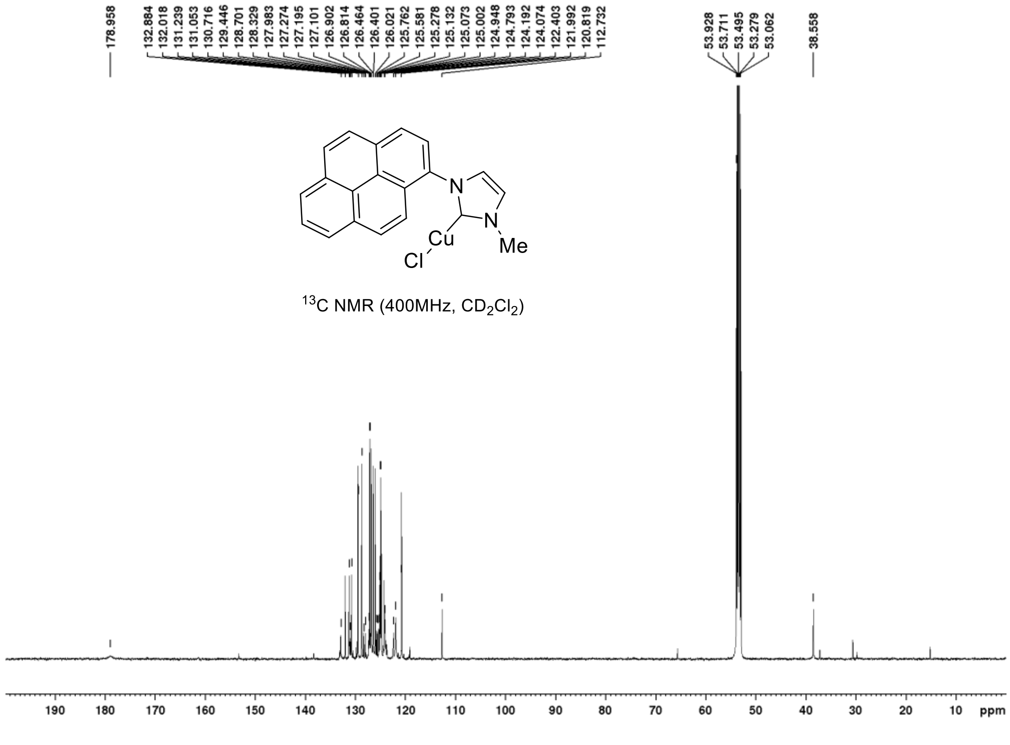

Figure S8. ^{13}C NMR of complex **3** in CD_2Cl_2

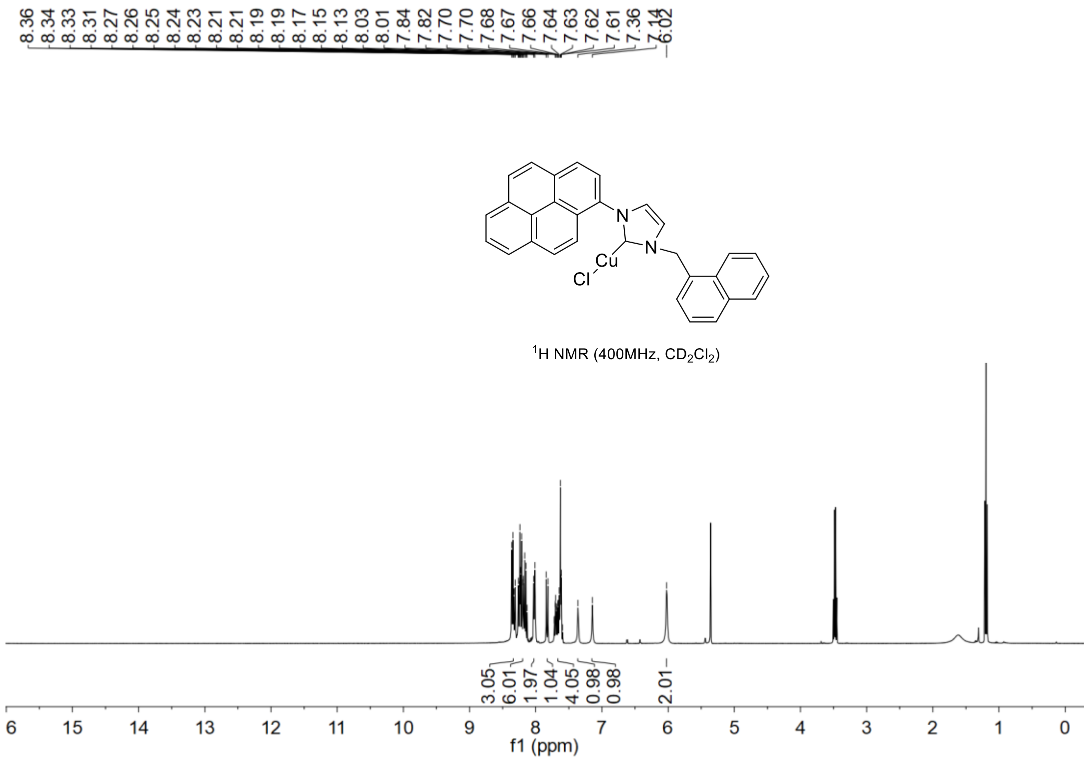

Figure S9. ^{1}H NMR of complex **4** in CD_2Cl_2

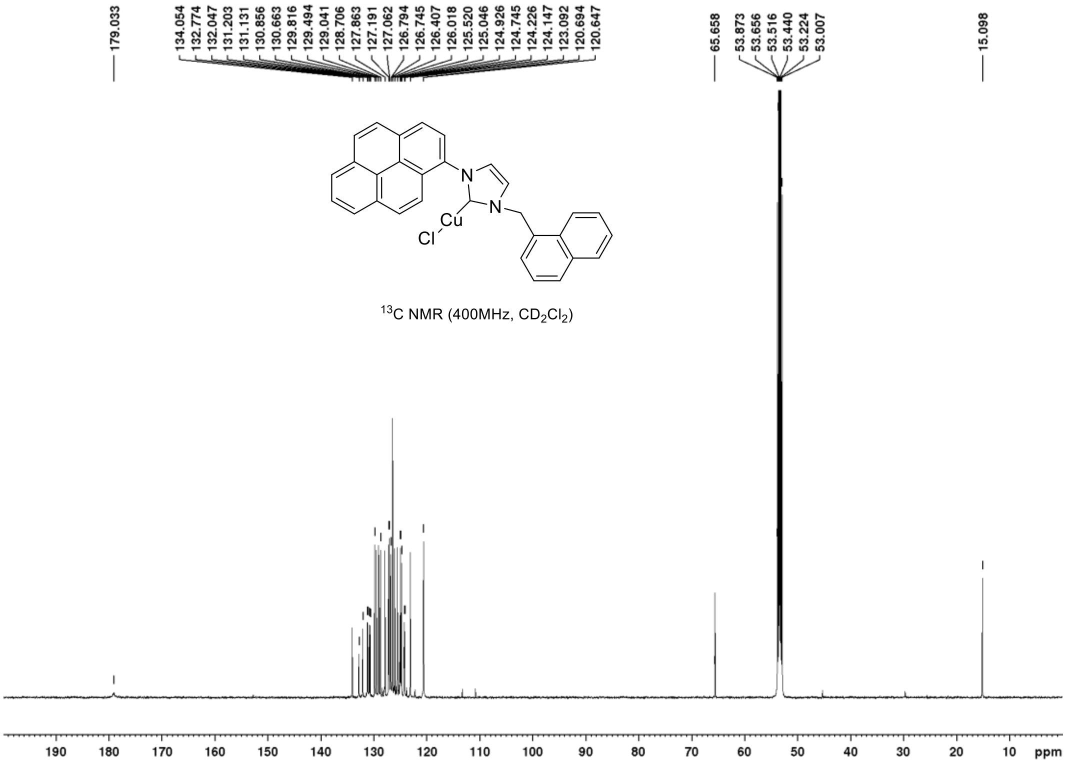

Figure S10. ^{13}C NMR of complex **4** in CD_2Cl_2

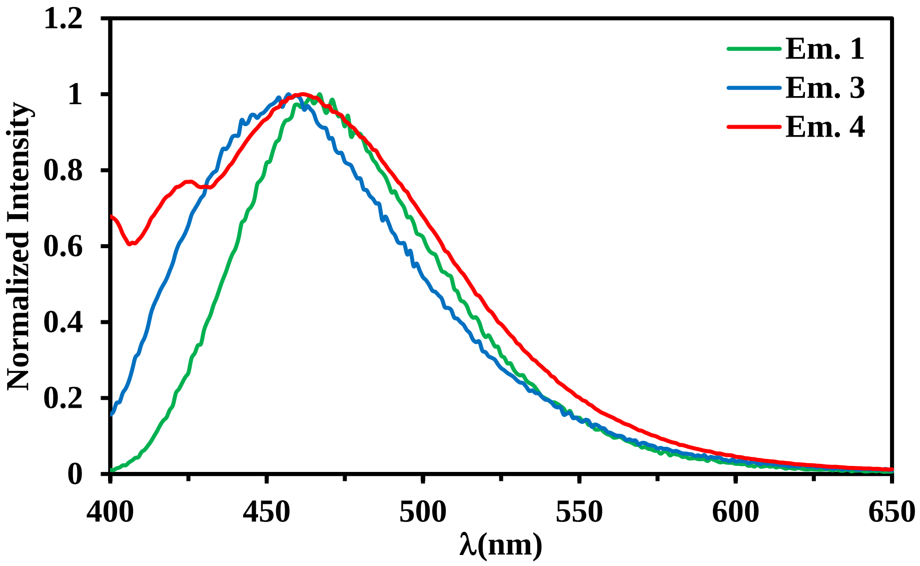

Figure S11. Normalized emission spectra of ligand **1** and complexes **3-4** in solid-state at rt.

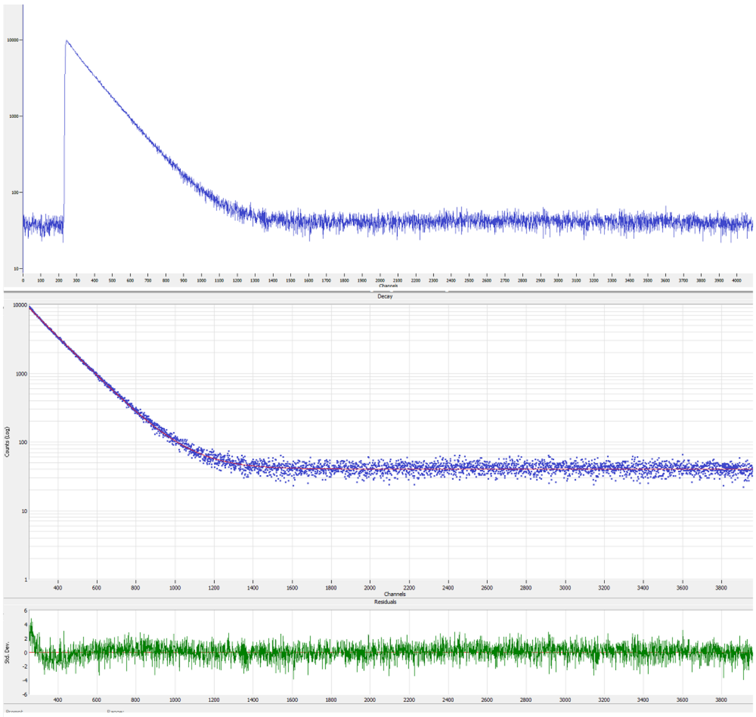

Figure S12. Emission decay (blue), best fit (red) and residuals (green) of **1** in CH_2Cl_2 at rt; $\chi^2 = 1.123$.

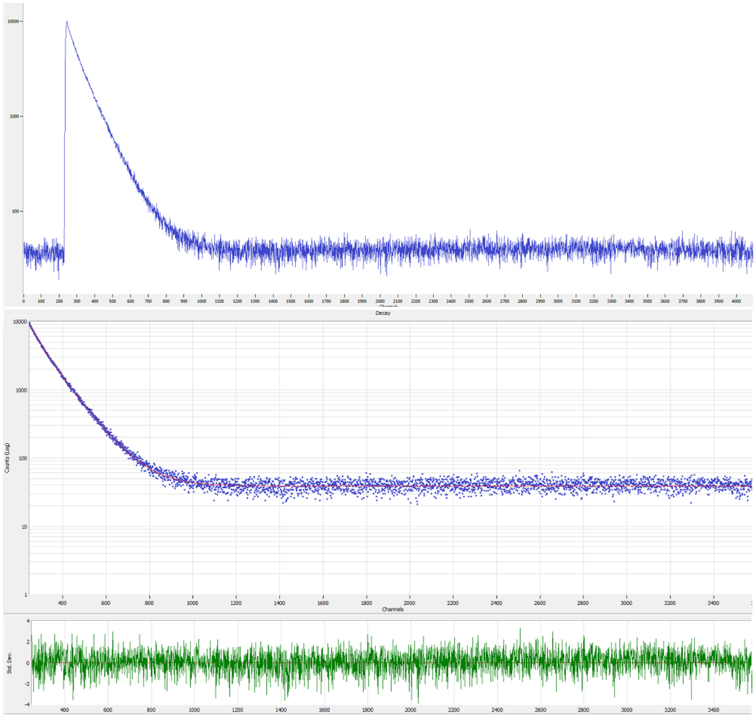

Figure S13. Emission decay (blue), best fit (red) and residuals (green) of **3** in CH_2Cl_2 at rt; χ^2 = 1.050.

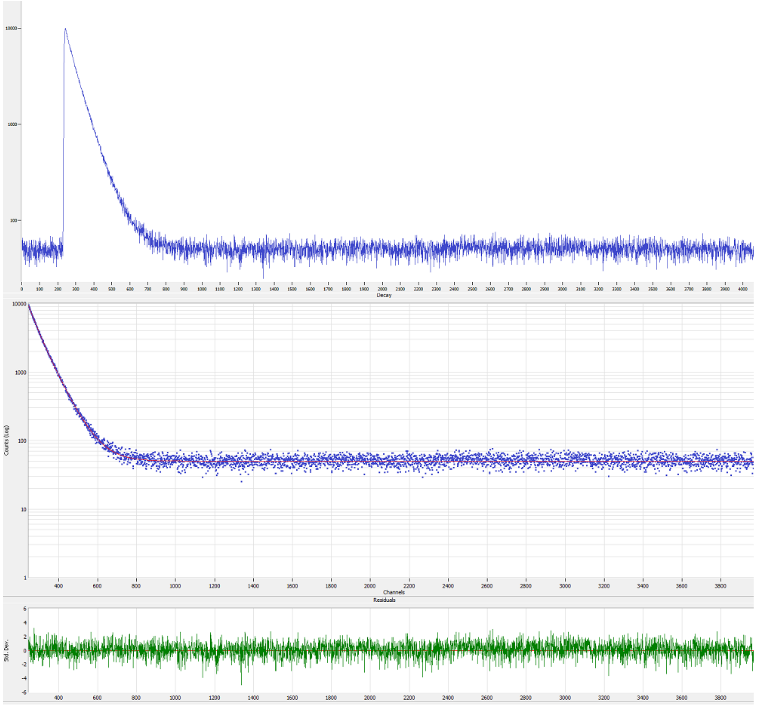

Figure S14. Emission decay (blue), best fit (red) and residuals (green) of **4** in CH_2Cl_2 at rt; χ^2 = 1.056.

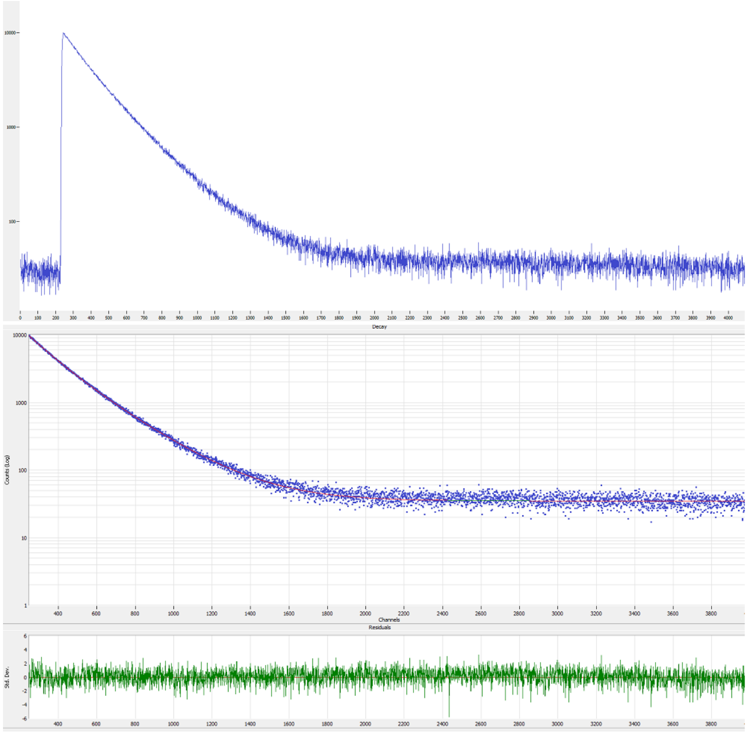

Figure S15. Emission decay (blue), best fit (red) and residuals (green) of **1** in solid-state at rt; χ^2 = 1.084.

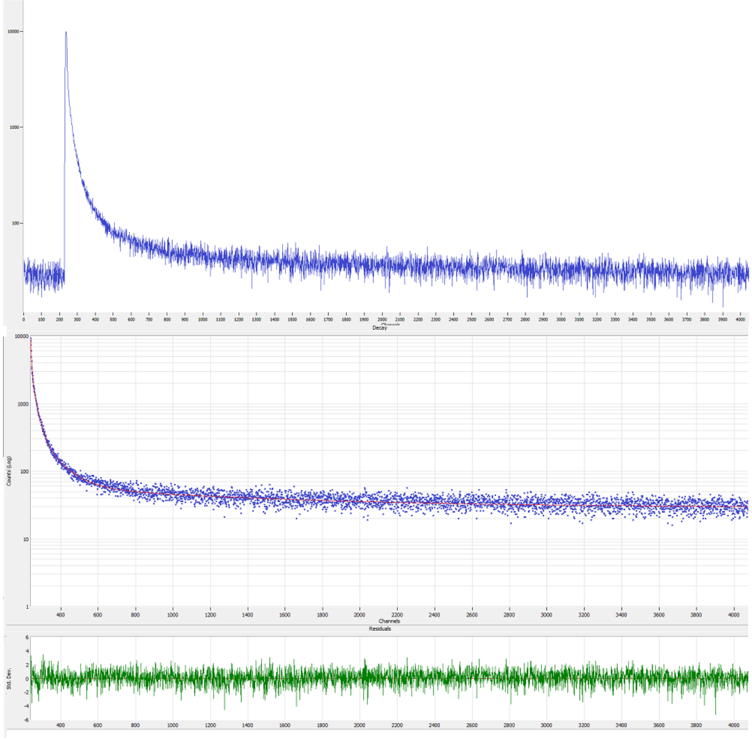

Figure S16. Emission decay (blue), best fit (red) and residuals (green) of **3** in solid-state at rt; χ^2 = 1.061.

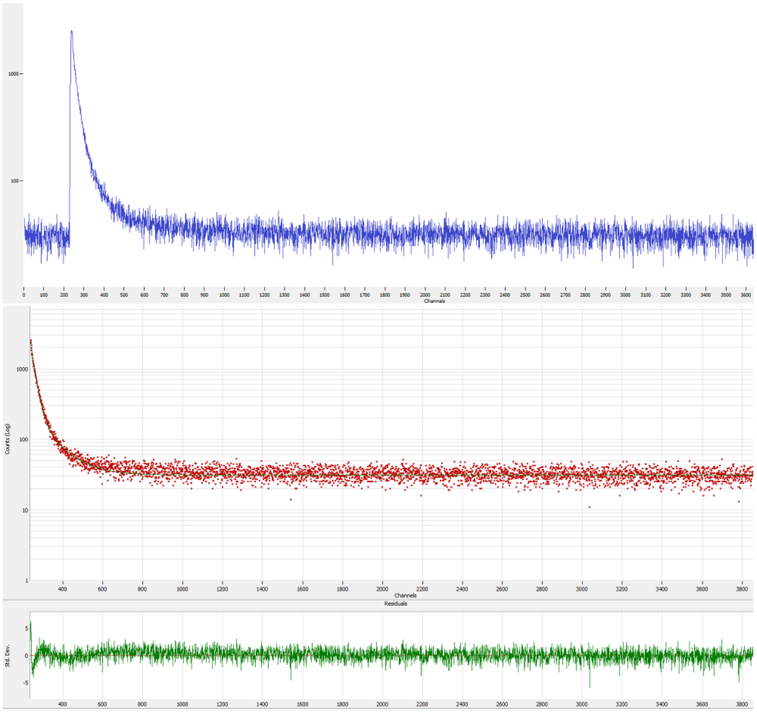

Figure S17. Emission decay (blue), best fit (red) and residuals (green) of **4** in solid-state at rt; χ^2 = 1.150.
